# *Actinomyces gerencseriae* hip prosthesis infection: a case report

**DOI:** 10.1186/s13256-015-0704-7

**Published:** 2015-09-28

**Authors:** Grégory Dubourg, Marion Delord, Frédérique Gouriet, Pierre-Edouard Fournier, Michel Drancourt

**Affiliations:** 1 Aix Marseille Université, URMITE, UM63, CNRS 7278, IRD 198, Inserm 1095, 13005 Marseille, France; 2 Pôle de Maladies Infectieuses, Hôpital de la Timone, Assistance Publique Hôpitaux de Marseille, Marseille, France; 3 Pôle de Maladies Infectieuses, Hôpital Nord, Assistance Publique Hôpitaux de Marseille, Marseille, France; 4 Unité de Recherche sur les Maladies Infectieuses et Tropicales Emergentes, Faculté de Médecine, 27, Boulevard Jean Moulin, 13385 Marseille Cedex 5, France

**Keywords:** *Actinomyces gerencseriae*, Infection, Orthopedic device, Hip prosthesis

## Abstract

**Introduction:**

*Actinomyces* bacteria are part of the human oropharyngeal microbiota. They have been associated with abdominal, cervicofacial and thoracic infections and a few cases of joint infections have also been described. In particular, *Actinomyces gerencseriae,* formerly described as *Actinomyces israelii* serovar II, has rarely been associated with human infections, mostly involving cervicofacial lesions and periodontal diseases. Here, we report one case of hip prosthesis infection due to *A. gerencseriae.*

**Case presentation:**

A 72-year-old Caucasian male developed an inflammatory collection on the outside of the right thigh where a hip prosthesis had been implanted for 11 years. Culturing a fluid sample from the collection puncture found *Staphylococcus hominis* and a Gram-positive bacillus unidentified by matrix-assisted laser desorption ionization time-of-flight mass-spectrometry (MALDI-TOF). Sequencing the 16S rRNA gene amplified from both the specimen and the isolate identified *A. gerencseriae*. Treatment adjusted with amoxicillin and trimethropim-sulfamethoxazole cured the infection.

**Conclusion:**

The recently described *A. gerencseriae* has rarely been involved in human infections. We report the first case of *A. gerencseriae* joint infection in a hip prosthesis.

## Introduction

*Actinomyces* bacteria are commensal members of the of oropharynx [1], digestive tract [2] and urogenital tract microbiota [3]. As pathogens, they are responsible for cervicofacial lesions [4], abdominopelvic infections [5] and respiratory tract infections [6]. *Actinomyces* bacteria have rarely been reported as being responsible for central nervous system (CNS) infections, skin infections [7] and bone and joint infections [7]. In this genus, *Actinomyces israelii* serovar II has been reclassified as *Actinomyces gerencseriae,* a commensal member of the human oral flora [8]; being further associated with cervicofacial infections [4], dental diseases [9, 10], in cases of osteoradionecrosis [11], but very rarely causing infection at other sites [12].

Here, we describe the first case of hip prosthesis infection due to this microorganism.

## Case presentation

A 72-year-old Caucasian male was diagnosed with an infected periprosthetic hematoma of the right hip. His medical history included bilateral osteoarthritis cured by the implantation of a right hip prosthesis 11 years previously and a left hip prosthesis four years previously, along with three myocardial infarctions followed by the implantation of ten coronary artery stents and the recent implantation of an implantable cardiac defibrillator (ICD) and consecutive warfarin treatment. Overdosage of the latter drug caused a right iliopsoas hematoma. Over the following three months, the patient presented with Guillain-Barré syndrome, which rapidly resolved after the administration of immunoglobulins, and angiocholitis cured by the administration of amoxicillin-clavulanate.

**Fig. 1:**
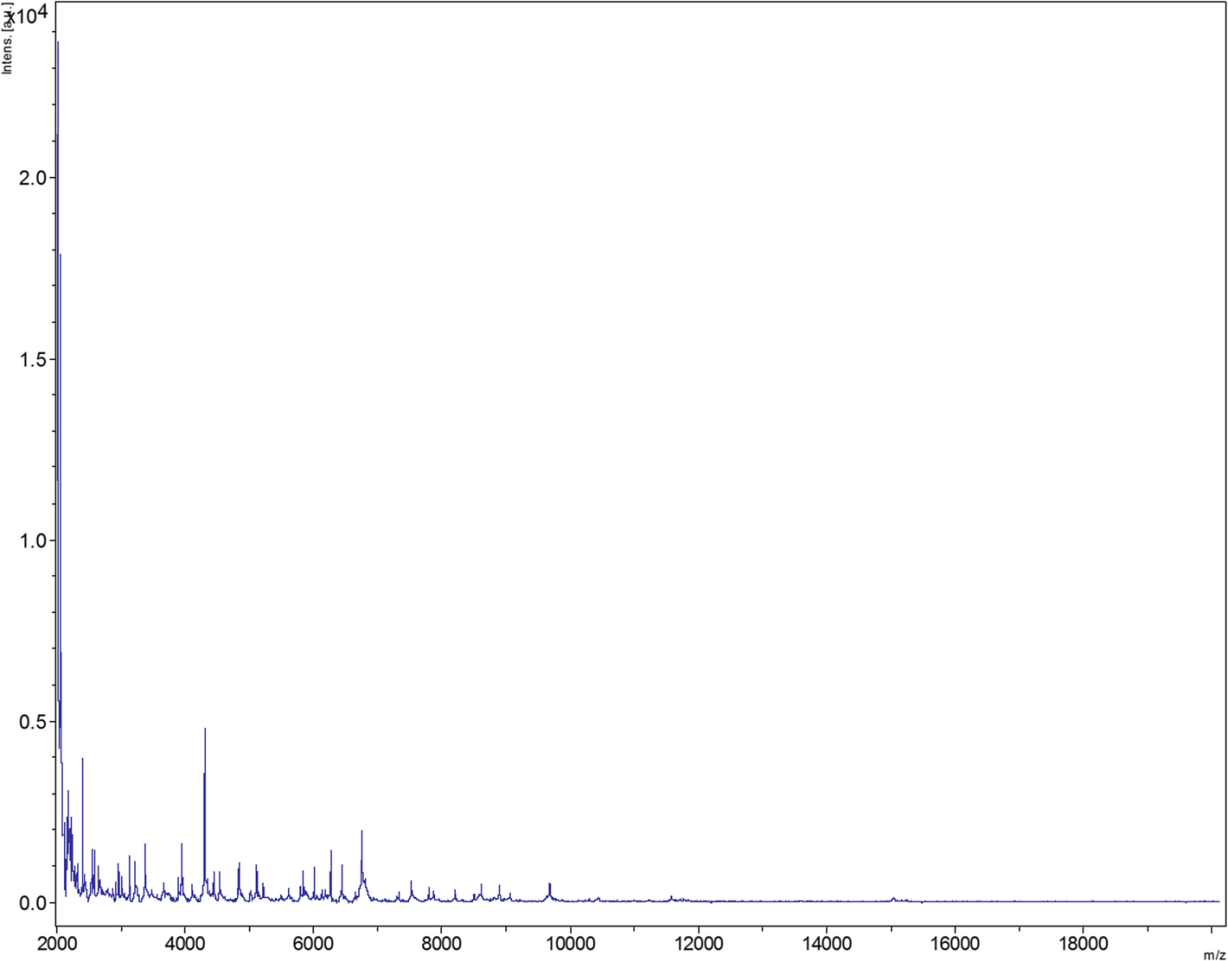
Reference mass spectrum from *A. genrencseriae* strain URMITE (= CSUR P1401). Spectra from 12 individual colonies were compared and a reference spectrum was generated

**Fig. 2:**
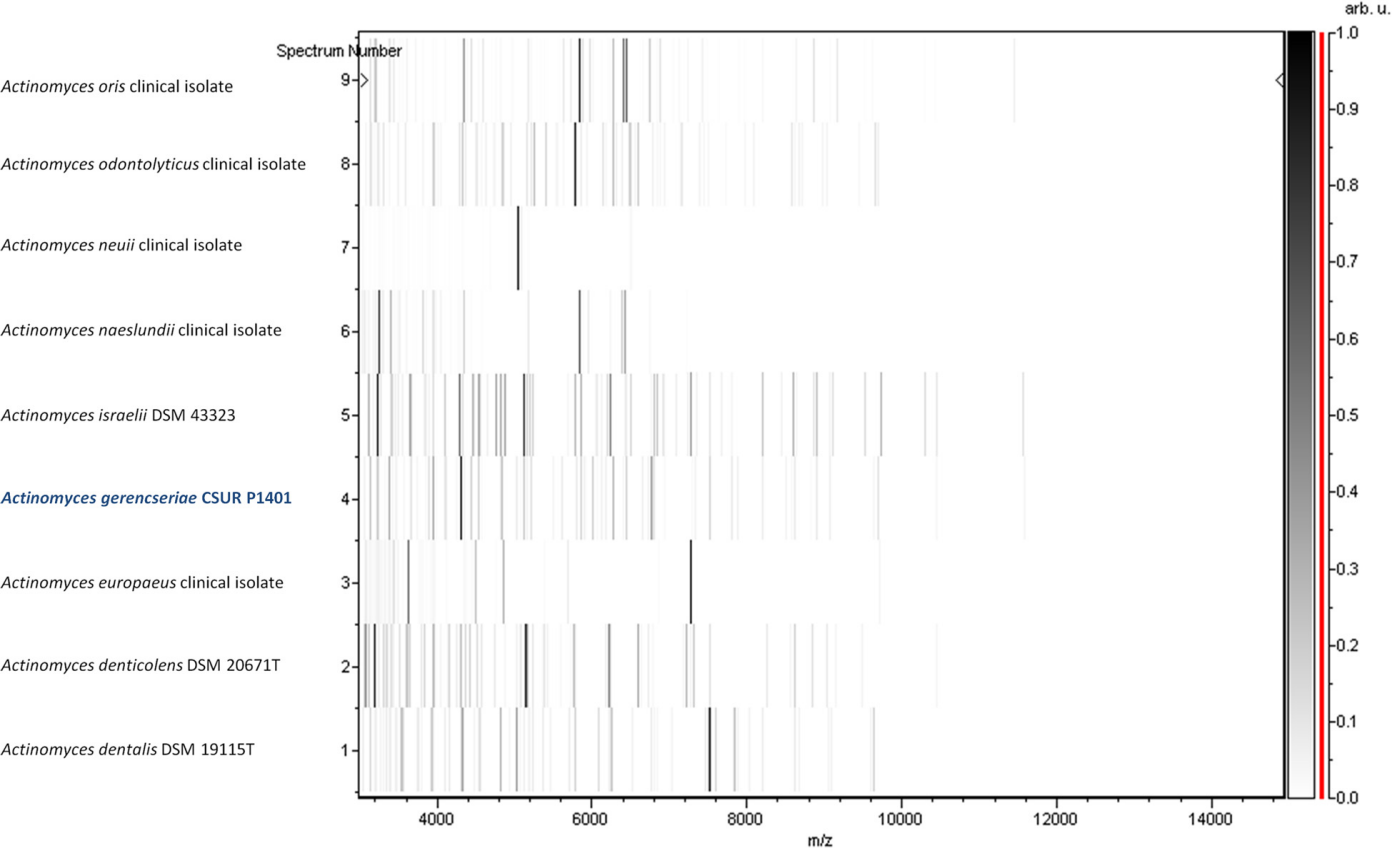
Gel view comparing *A. genrencseriae* strain URMITE (= CSUR P1401). The gel view displays the raw spectra of loaded spectrum files arranged in a pseudo-gel like look. The x-axis records the m/z value. The left y-axis displays the running spectrum number originating from subsequent spectra loading. Peak intensity is expressed by a greyscale code. The color bar and the right y-axis indicate the relation between the peak color displayed and peak intensity in arbitrary units. Displayed species are indicated on the left, while the URMITE strain is highlighted as blue

At the same time, he was diagnosed with fistulization of the infected iliopsoas hematoma on the outside of the right thigh, which had been neglected in view of other intercurrent medical episodes. This was subsequently treated for eight weeks by amoxicillin-clavulanate and fusidic acid without microbiological documentation. Four months later, the right hip collection persisted and an incision with drainage was conducted. Several PCR tests, including 16S rRNA gene amplification [13] performed on the sampled fluid, were negative and the standard culture was sterile. At that time, the white blood cell count was normal at 8.4×10^9^/L (the neutrophil count was 6.2×10^9^/L) and the platelet count was 246×10^9^/L. The erythrocyte sedimentation rate was elevated at 82mm/hour. A second specimen, sampled eight weeks later, grew two types of colonies on Columbia agar with 5% sheep blood (bioMérieux, Marne la Coquette, France) incubated at 37°C in a 5% CO_2_-enriched and anaerobic atmosphere. Matrix-assisted laser desorption ionization time-of-flight mass-spectrometry (MALDI-TOF-MS) [14], that allows bacterial identification through their mass spectra, identified one colony as *Staphylococcus hominis* with an identification score of 2.02. However, MALDI-TOF-MS identification of the second colony failed. This Gram-positive bacillus was then identified by PCR-sequencing of the 16S rRNA gene as previously described [13]. A 1,486-bp sequence (GenBank LN624398) yielded 99.2% similarity with *A. gerencseriae* (Genbank X80414) using NCBI BLAST (http://www.ncbi.nlm.nih.gov). This isolate was deposited in the Collection de Souches de l’Unité des Rickettsies (=CSUR P1401). This *A. gerencseriae* MALDI-TOF-MS spectrum was subsequently added to the database (Fig. 1) in order to be specifically compared to eight other *Actinomyces* spectra available in the database (Fig. 2). Further amplification and sequencing of the 16S rRNA gene directly on the sampled fluid yielded a 999-bp sequence exhibiting 98.9% sequence similarity with *A. gerencseriae* (Genbank NR029280) and 99.7% sequence similarity with that of the isolate (GenBank LN624398). The antibiotic regimen was adapted with amoxicillin and trimethoprim-sulfamethoxazole after the minimum inhibitory concentrations had been measured at 0.023mg/L and <1mg/L, respectively. This treatment was stopped three weeks later due to kidney failure. Further microbiological investigation found *Staphylococcus aureus* and ofloxacin combined with rifampicin was finally prescribed.

This patient presented a mixed infection of a hip prosthesis with *A. gerencseriae* being one of the three documented organisms. The presence of this organism was definitively confirmed by two different techniques. Thus, direct 16S rRNA gene amplification in a puncture product strengthened the culture results, excluding laboratory contamination and indicating that the microorganism was indeed present in the collected specimen. Moreover, *A. gerencseriae* [8] inhabits the human oral microbiota [15] but not human skin, rendering the probability of per-operative contamination highly improbable. Also, *A. genrencseriae* is not known as a laboratory contaminant and we had no other case documented in our laboratory. Therefore, we interpreted *A. genrencseriae* as being part of a mixed hip prosthesis infection in this patient.

In this case, *A. genrencseriae* was firmly identified on the basis of two independent 16S rRNA gene PCR amplifications and sequencing which yielded the identical partial 16S rRNA gene sequence. However, MALDI-TOF-MS identification failed since *A. genrencseriae* was not incorporated into the commercial database we used; accordingly, we added its spectrum in order to allow for its subsequent identification by MALDI-TOF-MS.

Although the species *A. genrencseriae* has been known for 25 years, it has been implicated only rarely in infections, mainly head and neck infections including periodontal disease [9], cervicofacial infected lesions [4], mandibular osteoradionecrosis [12], ulcerative gingivitis (NUG) and oral inflammatory lesions [10]. Involvement in eye infections and chronic granulomatous diseases has also been reported [12].

## Conclusions

*A. genrencseriae* is a fastidious organism [15] whose identification still requires 16S rRNA gene sequencing, pending incorporation of the appropriate spectrum in MALDI-TOF-MS databases. These particularities explain why very few cases of *A. genrencseriae* infection have been reported. The case reported here indicates that *A. genrencseriae* infections are by no means limited to head and neck infections.

## Consent

Written informed consent was obtained from the patient for publication of this case report and any accompanying images. A copy of the written consent is available for review by the Editor-in-Chief of this journal.
